# Mortality across the spectrum of hemoglobin level in patients undergoing surgical coronary revascularization

**DOI:** 10.1002/clc.24004

**Published:** 2023-03-23

**Authors:** Amirmohammad Khalaji, Ali Ajam, Ali Sheikhy, Amir Hossein Behnoush, Aida Fallahzadeh, Jamshid Bagheri, Soheil Mansourian, Shahram Momtahen, Farzad Masoudkabir, Kaveh Hosseini

**Affiliations:** ^1^ Tehran Heart Center, Cardiovascular Diseases Research Institute Tehran University of Medical Sciences Tehran Iran; ^2^ Cardiac Primary Prevention Research Center, Cardiovascular Diseases Research Institute Tehran University of Medical Sciences Tehran Iran; ^3^ Non‐Communicable Diseases Research Center, Endocrinology and Metabolism Population Sciences Institute Tehran University of Medical Sciences Tehran Iran; ^4^ School of Medicine Tehran University of Medical Sciences Tehran Iran

**Keywords:** anemia, coronary artery bypass, hemoglobin, mortality

## Abstract

**Background:**

Preoperative hemoglobin (Hb) level is a predictor of in‐hospital and midterm mortality in patients undergoing coronary artery bypass grafting surgery (CABG). However, the debate about the different hazards across Hb levels and sex differences in outcome occurrence is still on the table.

**Methods:**

This is a registry‐based serial cross‐sectional study at Tehran Heart Center. Nonanemic patients who underwent CABG with complete follow‐up data were included. The Restricted Cubic Splines (RCS) in the Cox model was used to calculate the sex‐specific correlation between in‐hospital, 6‐month, and 1‐year mortalities and normal Hb levels using odds ratio for the in‐hospital and hazard ratios for 6‐month and 1‐year mortality, adjusted for all possible confounders.

**Results:**

From 2005 to 2016, a total of 13,557 patients were included, of which 134 had in‐hospital mortality as our primary outcome. Preoperative Hb levels were significantly lower in the deceased. Moreover, dead patients had significantly higher rates of diabetes and hypertension, while lower ejection fraction. Cut‐offs for reference Hb level were higher for males compared with females. The correlation between Hb level and in‐hospital mortality was nearly U‐shaped. Quantitatively, Hb of ≥15.62 and ≤13.25 g/dL for men and that of ≥14.92 and ≤13.4 g/dL for women tended to be associated with higher in‐hospital mortality.

**Conclusions:**

The association between preoperative Hb level and in‐hospital mortality differs in men and women and does not follow a linear correlation among nonanemic patients. Both low and high numbers in the Hb level spectrum are at greater risk.

## Graphical abstract

In this study conducted at Tehran Heart Center, we investigated the association between preoperative hemoglobin levels and mortality in 13,557 patients undergoing coronary artery bypass grafting (CABG) surgery. In‐hospital mortality occurred in 134 patients. Hemoglobin cutoffs were higher for men, compared with women, and the correlation between hemoglobin levels and in‐hospital mortality was almost U‐shaped. In conclusion, both high and low hemoglobin levels accounted for higher mortality, which can have clinical implications.

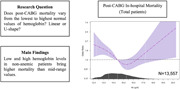

## 1 INTRODUCTION

The prevalence of cardiovascular diseases (CVDs) has doubled from 271 million cases in 1990 to 523 million in 2019, while the number of cardiovascular‐related deaths elevated by 50% from 12.1 million in 1990, reaching 18.6 million in 2019.^1^ Coronary artery bypass grafting (CABG) surgery is a lifesaving procedure in severe coronary artery disease.^2^ Besides the importance of procedure techniques, baseline and preoperative features and comorbidities determine its success rate, in addition to short‐term and long‐term outcomes.^3^, ^4^, ^5^, ^6^, ^7^, ^8^, ^9^

Anemia can cause postoperative complications such as arrhythmia, hypoxemia, and heart failure; if left untreated.^10^ The major adverse cardiac and cerebrovascular events rate in patients undergoing coronary artery bypass grafting with preoperative anemia is higher than in patients with normal Hb concentration.^11^, ^12^, ^13^, ^14^, ^15^ Hemoglobin (Hb) level variation may affect in‐hospital and midterm mortality in anemic or even nonanemic patients undergoing CABG.^12^ Although the association between preoperative anemia (based on Hb level) and post‐CABG mortality has been well established before,^11^, ^12^, ^13^, ^14^, ^15^ the outcomes were not evaluated across the spectrum of Hb in nonanemic patients.

Therefore, in this study, we aimed to evaluate the association between baseline normal Hb levels, and post‐CABG in‐hospital and midterm mortality. In addition, we determined the sex‐specific prevalence of all‐cause mortality in patients with different levels of Hb.

## 2 METHODS

### 2.1 Study design and population

This is a registry‐based serial cross‐sectional study conducted between 2005 and 2016 at Tehran Heart Center (THC). THC is a major tertiary referral center in Tehran, Iran, dedicated to the treatment of heart diseases.^16^ All the patients who underwent isolated CABG at our center were eligible to be included. We excluded anemic patients and ones without preoperative Hb concentration data. Anemia was defined using World Health Organization (WHO) criteria as Hb below 13 g/dL in men and 12 g/dL in women.^17^ Procedures were all performed by expert cardiovascular surgeons with more than 200 operations before the current study. Patients were treated under official guidelines practiced in THC, and no trial intervention was performed. We conducted this study under the declaration of Helsinki, and it was approved by the ethical committee of THC (IR.TUMS.THC.REC.1401.049) in June 2022.^18^ The “informed consent waiver” was obtained from the ethical board of THC due to the deletion of patients' names except for the corresponding author and database chief.

### 2.2 Baseline assessment

Patients' Hb levels were recorded before the operation as a part of the complete blood count (CBC) test, reported in grams per deciliter (g/dL). Demographics of the patients, including age, sex, weight, height, smoking status, opium consumption, and family history of coronary artery disease, were obtained before admission. Past medical history of previous myocardial infarction (MI), previous CABG, previous percutaneous coronary intervention (PCI), cerebrovascular accident (CVA), chronic obstructive pulmonary disease (COPD), and complete drug history were also recorded. Fasting blood glucose (FBS), low‐density lipoprotein cholesterol (LDL‐C), high‐density lipoprotein cholesterol (HDL‐C), total cholesterol, and triglycerides were measured during hospitalization. Finally, left ventricular ejection fraction (LVEF) was measured using echocardiography, and used as a risk estimator for surgery. Total intensive care unit (ICU) time, total ventilation time, and total admission days were recorded in our databank as postoperative variables.

### 2.3 Follow‐up protocol and study endpoints

Patients were evaluated 6 months and 1 year after CABG and then annually after surgery by direct clinic visits. Patients who did not attend the clinic were followed by telephone interviews. The primary endpoint was in‐hospital mortality while 6‐month and 1‐year mortality were secondary endpoints in the current study.

### 2.4 Statistical analysis

Normally and non‐normally distributed continuous variables were presented as mean with standard deviation (SD) and median with 25th and 75th percentiles (interquartile range [IQR] boundaries), respectively. The Kolmogorov–Smirnov test was used to test for the data normality.^19^ Variables were compared between the dead and survived groups utilizing the student's *t* test for normally distributed and Mann–Whitney *U* test for skewed distributed variables. The *χ* ^2^ test was applied to compare categorical variables.

To eliminate the gender effect on mortality, data were stratified into “male” and “female.” For the in‐hospital mortality, a logistic regression model calculating odds ratio (OR) with its 95% confidence interval was used. The adjusted Cox proportional hazards model was used to evaluate the Hb effect on in‐hospital and mid‐term mortality. Hb relationship with in‐hospital, 6 months, and 1‐year mortality hazards were estimated by entering Hb as a continuous variable in the adjusted model. The Restricted Cubic Splines (RCS) in the Cox model allow prospecting for a nonlinear relationship of Hb level with the “Odds Ratio” of in‐hospital mortality and “Hazard ratio” of 6 months and 1‐year mortality, estimated from the Cox regression model adjusted for all possible confounders. We applied five knots (df = 4), applied at the 5th, 25th, 50th, 75th, and 95th percentile of Hb levels. A cutoff point was identified to investigate the independent prognostic role of Hb level using the optimal equal‐hazard ratio (HR) method, which selects optimal cut‐points of continuous predictors that have U‐shaped relationships with log(λ) in survival analysis. The statistical details are beyond the scope of this study and are mentioned elsewhere.^20^ Data analysis was performed using R version 4.0.4 and the *p* value of <.05 was considered statistically significant.

## 3 RESULTS

### 3.1 Baseline characteristics

**Figure 1:**
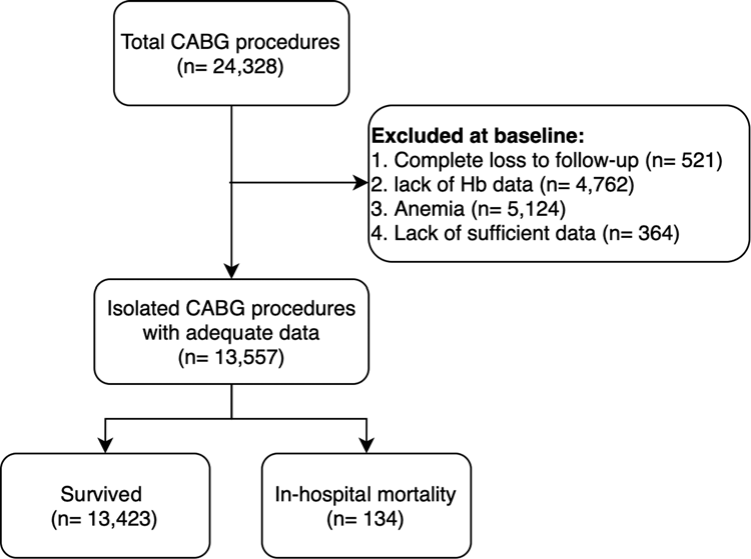
Diagram of inclusion and exclusion criteria for patients included in the study.

Of the 24,328 recorded patients, we excluded 10,771 patients due to various reasons, including having anemia, as described comprehensively in Figure 1. Among 13,557 evaluated patients, the in‐hospital mortality was 0.99% (134 cases). Males had a 0.74% mortality rate, while this was 1.77% for females, pointing to a significant increase in them (*p* < .001). Lower preoperative LVEF and lower estimated glomerular filtration rate (eGFR), in addition to higher diabetes, hypertension, and renal failure rates, were also recorded in dead patients. The baseline characteristics of patients are summarized in Table 1.

**Table 1:** Baseline characteristics in survived and dead patients.

		In‐hospital		
		Survived (*n* = 13,423)	Dead (*n* = 134)	*p* value
Hb		14.40 (14.40–14.50)	13.80 (13.50–14.10)	**.001**
Age		66.73 ± 9.64	72.50 ± 9.74	**<.001**
Gender				
Female		23.9%	43.3%	**<.001**
		(3209)	(58)	
Male		76.1%	56.7%	
		(10214)	(76)	
BMI (kg/m^2^)		27.35 ± 0.04	27.84 ± 0.43	.172
Preoperative EF (%)		50.0 (50.0–52.0)	42.3 (40.0–45.0)	**<.001**
eGFR (mL/min/1.73 m^2^)		86.45 (67.10–107.98)	81.17 (62.96–103.91)]	**.002**
Graft number				
1		1.8%	2.2%	
		(245)	(3)	
2		11.4%	14.9%	
		(1526)	(20)	
3		38.5%	43.3%	.331
		(5163)	(58)	
4		39.3%	32.8%	
		(5278)	(44)	
5+		9.0%	6.7%	
		(1211)	(9)	
Diabetes		37.1%	49.3%	**.004**
		(4983)	(66)	
Hypertension		51.9%	73.1%	**<.001**
		(6967)	(98)	
Dyslipidemia		53.4%	54.5%	.804
		(7168)	(73)	
Current Cigarette smoker		18.8%	17.9%	.782
		(2530)	(24)	
Renal Failure		0.8%	5.2%	**<.001**
		(111)	(7)	
COPD		3.5%	6.0%	.114
		(464)	(8)	
Cerebrovascular accident		6.4%	10.4%	.055
		(854)	(14)	
Off‐pump surgery		9.3%	9.0%	.899
		(1234)	(12)	
Cross‐clamp time (min)		39.00 (39.00–40.00)	41.00 (35.00–48.00)	.117

*Note*: Data are presented as median (interquartile range) or mean ± standard deviation for continuous variables and % (number) for categorical variables. Bold *p*‐values represent statistically significant differences.

Abbreviations: BMI, body mass index; COPD, chronic obstructive pulmonary disease; EF, ejection fraction; eGFR, estimated glomerular filtration rate; Hb, hemoglobin.

### 3.2 Hb cut‐offs

As the normal Hb range for men and women differs, the analyses were divided based on patients' sex. Table 2 shows the reference range of Hb levels calculated by RCS for determining the HR for males and females. Cut‐off points for in‐hospital mortality were approximately equal for men and women (13.25–15.62; 13.40–14.92; respectively). Similarly, for 6‐months and 1‐year mortality, the estimated range was wider in men compared with women (6 months: 13.20–16.40 for men and 12.37–13.60 for women; 1 year: 13.20–16.40 for men and 12.39–13.60 for women).

**Table 2:** Reference ranges of hemoglobin (g/dL) for odds or hazard ratios determined by Restricted cubic spline (RCS).

Male	Female
In‐hospital mortality^a^	
13.25−15.62	13.4−14.92
Six‐month mortality^b^	
13.20−16.40	12.37−13.60
One‐year mortality^b^	
13.20−16.40	12.39−13.60

Abbreviation: RCS, Restricted Cubic Splines.

^a^ Reference range of odds ratio determined by RCS.

^b^ Reference range of hazard ratio determined by RCS.

### 3.3 Main outcomes

#### 3.3.1 In‐hospital mortality

**Figure 2:**
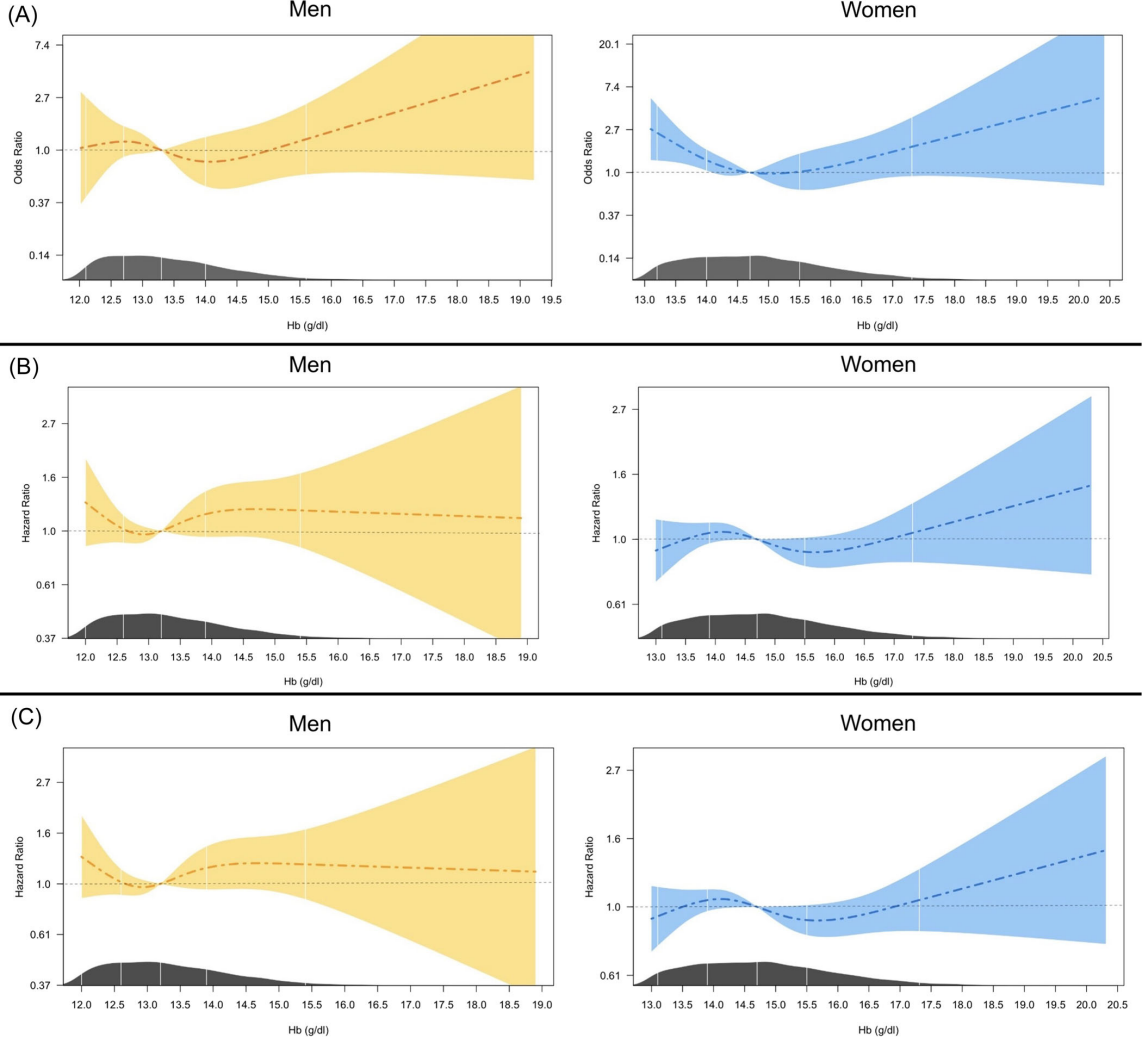
Restricted cubic spline plot for odds ratio (OR) or hazard ratio (HR) with 95% confidence interval in different levels of hemoglobin in men and women for (A) in‐hospital mortality (OR); (B) 6‐month mortality (HR); and (C) one‐year mortality (HR).

Calculated gender‐specific ORs for mortality based on baseline Hb levels are presented in Table 3. A nearly U‐shaped correlation between Hb level and in‐hospital mortality is depicted in Figure 2A. Men with Hb levels higher than 15.62 g/dL and women with higher than 14.92 g/dL had a tendency for higher in‐hospital mortality compared with the reference range of Hb (men: OR = 1.05 [95% confidence interval [CI]: 0.62–1.92], *p* = .925; women: OR = 2.06 [95% CI: 0.93–5.67], *p* = .073). Moreover, although not significant, a higher incidence of in‐hospital mortality was observed with Hb lower than 13.4 g/dL in women (OR = 1.51 [95% CI: 0.82–2.94], *p* = 0.180). However, men with Hb below 13.25 g/dL had significantly higher mortality than the reference range (OR = 2.11 [95% CI: 1.10–4.05], *p* = .025).

**Table 3:** Adjusted odds or hazard ratios for mortality in men and women.

Male		Female	
*In‐hospital mortality* a			
Hb ≤ 13.25	Hb ≥ 15.62	Hb ≤ 13.4	Hb ≥ 14.92
2.11 [1.10–4.05] *p* = .025	1.05 [0.62–1.92] *p* = .925	1.51 [0.82–2.94] *p* = .180	2.06 [0.93–5.67] *p* = .073
*Six‐month mortality* b			
Hb ≤ 13.20	Hb ≥ 16.40	Hb ≤ 12.37	Hb ≥ 13.60
0.90 [0.70–1.17] *p* = .443	1.02 [0.79–1.30] *p* = .910	**1.51 [1.09–2.09] *p*** = **.014**	1.22 [0.99–1.60] *p* = .052
*One‐year mortality* b			
Hb ≤ 13.20	Hb ≥ 16.4	Hb ≤ 12.39	Hb ≥ 13.60
0.92 [0.71–1.19] *p* = .410	1.05 [0.81–1.32] *p* = .772	**1.51 [1.09–2.09] *p*** = **0.014**	**1.24 [1.02–1.60] *p*** = **.041**

*Note*: Data are presented as adjusted hazard ratios (95% confidence interval). Adjustment for: age, body mass index, diabetes, hypertension, chronic obstructive pulmonary disease, estimated glomerular filtration rate, family history of cardiac disease, cerebrovascular accidents, preoperation percutaneous coronary intervention, off‐pump surgery, and urgent/emergent operation.

Abbreviations: HR, hazard ratio; Hb, hemoglobin.

^a^ Reference range of odds ratio determined by RCS.

^b^ Reference range of hazard ratio determined by RCS.

#### 3.3.2 Six‐month and 1‐year mortality

Mortality HRs were almost identical in 6‐month and 1‐year follow‐ups (Table 3 and Figure 2). In women, Hb below the calculated reference range was correlated with higher 6‐month and 1‐year mortality (HR = 1.51 [95% CI: 1.09–2.09] and *p* = .014 for both). This was also the case for Hb levels higher than 13.6 g/dL, however, HR was only significant for 1‐year mortality (HR = 1.24 [95% CI: 1.02–1.60], *p* = .041). Another difference was that the nadir of the mortality HRs moved up to higher Hb levels in men, but it decreased in women compared with in‐hospital results. Also, compared with in‐hospital mortality, 6‐month, and 1‐year estimated HRs in women tend to decrease in higher Hb levels (Figure 2A vs. Figure 2B–C).

## 4 DISCUSSION

The findings of the present study suggest that higher preoperative Hb levels have a clear tendency toward higher mortality in both men and women. In addition, patients with lower limits of normal Hb levels were at greater risk compared with mid‐normal values. Although anemia was thoroughly discussed in the literature as an important risk factor for in‐hospital mortality after CABG,^13^, ^14^, ^15^, ^21^, ^22^, ^23^, ^24^, ^25^, ^26^, ^27^, ^28^, ^29^, ^30^, ^31^ the difference in normal Hb levels was not evaluated before.

Our study found that there is a higher mortality in females, compared with males. This is in line with other studies assessing CABG mortality.^32^, ^33^ This can be justified by the higher rate of comorbidities, delay in diagnosis of cardiovascular diseases, and older age in women undergoing CABG.^34^, ^35^ However, as mentioned by Schmidt et al, this difference between men and women could be attributed to their characteristics and comorbidities which are mainly different.^32^ Therefore, preoperative patients' profile is significantly different between sexes, which can affect the outcomes of CABG.^30^ In 2021, Ripoll et al. concluded that associations between Hb levels and outcomes were distinct for females and males, with different spline knot points (13 and 14 g/dL, respectively). As reported, in females, each 1 g/dL decrease in Hb below 13 was associated with increased odds of acute kidney injury (AKI).^11^ Mechanistically, there is an increased risk of insufficient delivery of tissue oxygenation through lower Hb levels preoperatively which can independently be correlated with mortality, cerebral accident, and AKI, as mentioned.^36^, ^37^, ^38^ Moreover, patients with lower Hb levels might have limited ability to compensate through an increase in heart rate and stroke volume.^39^

Kullier et al. reported a dose‐dependent significant increase in adverse outcomes including mortality and cardiac complications, with decreasing Hb levels compared with patients with Hb>14.^24^ Van Straten et al. found that although anemia is a risk factor for early, defined as 30 days after surgery, and late mortality, while low preoperative Hb level is an independent predictor of late mortality after CABG. Hb levels of more than 14.5 g/dL in men and 13.5 g/dL in women were associated with both lower early and late mortality compared with normal and low Hb in this study,^31^ unfortunately, they did not evaluate the trend of HR in normal values. Ewila et al. also reported that preoperative Hb level could be an indicator of outcome after CABG. They noted that preoperative blood loss of more than 1 L was slightly higher in patients with Hb >15 g/dL compared with 15 g/dL > Hb > 12 g/dL. Higher blood loss in higher Hb levels may be the cause of higher in‐hospital mortality in our patients. Moreover, the percentage of patients with postoperative stay >7 days was lower in patients with normal Hb levels compared with the high‐Hb (>15 g/dL) and low‐Hb (<12 g/dL) groups,^23^ although the difference between high‐Hb group and normal Hb group was not significant. In this study, higher rates of bleeding and postoperative infection were observed in the low preoperative Hb level group which could be the main causes of longer hospital stay in this group. Overall, this emphasizes the fact that controlling Hb levels can lead to earlier discharge and lower costs for patients and healthcare systems.

Aside from enrolling nonanemic patients, major differences between our study compared with studies evaluating the effect of Hb level independent of anemia were the longer follow‐up period for mortality and gender‐specific reference range and results. We also estimated gender‐specific cutoffs of baseline Hb for post‐CABG mortality. Certainly, this concept is novel and other studies are required to elaborate further on this matter.

### 4.1 Limitations

We did our best to include and adjust all important confounding parameters. However, more data are needed to evaluate etiologic pathways in addition to other possible confounders which were not included. In assessing blood loss in the operation room, the number of transfused packed cells is among the important variables. The association between transfusion and post‐CABG mortality has been established before, and its effect is independent of the presence of preoperative anemia and Hb levels. To decrease the effect of this confounder, we enrolled only nonanemic patients in our study. Although not able to omit this factor and as reported before that most anemic patients who underwent CABG needed blood transfusion during surgery, our inclusion criteria could overcome this issue to some extent.

Mid‐ and long‐term outcomes are associated with much more parameters and cannot be justified merely with preoperative baseline characteristics. Unfortunately, we could not capture changes in risk factor profile after CABG; hence postdischarge results should be assessed with caution.^11^, ^22^, ^26^ Finally, the single‐center nature of our study is another limitation that should be taken into consideration. This warrants further similar studies in other settings to confirm the findings of the present study.

## 5 CONCLUSION

Simple one‐value cut‐offs in quantitative parameters are insufficient and sometimes misleading. Patients at the highest end of Hb levels and in lower limit normal showed a tendency toward higher in‐hospital mortality after CABG. Moreover, there is a clear gender difference in Hb cut‐offs and effects. This study was hypothesis‐generating and needs confirmation with a more detailed analysis in a larger sample size.

## AUTHOR CONTRIBUTIONS

*Study conception/data analysis/drafting the manuscript/revision*: Amirmohammad Khalaji, Ali Ajam, Ali Sheikhy, Amir Hossein Behnoush, Aida Fallahzadeh. *Drafting the manuscript/revision*: Jamshid Bagheri, Soheil Mansourian, Shahram Momtahen, Farzad Masoudkabir. S*tudy conception/drafting the manuscript/critical revision*: Kaveh Hosseini.

## CONFLICT OF INTEREST STATEMENT

The authors declare no conflict of interest.

## Data Availability

The data used in this study will be made available upon reasonable request from the corresponding author.
